# Life expectancy and disease burden in the Nordic countries: results from the Global Burden of Diseases, Injuries, and Risk Factors Study 2017

**DOI:** 10.1016/S2468-2667(19)30224-5

**Published:** 2019-11-20

**Authors:** Nordic Burden of Disease Collaborators, Ann Kristin Knudsen, Peter Allebeck, Mette C Tollånes, Jens Christoffer Skogen, Kim Moesgaard Iburg, John J. McGrath, Knud Juel, Emilie Elisabet Agardh, Johan Ärnlöv, Tone Bjørge, Juan J Carrero, Christopher R. Cederroth, Anne Elise Eggen, Ziad El-Khatib, Christian Lycke Ellingsen, Seyed-Mohammad Fereshtehnejad, Mika Gissler, Kishor Hadkhale, Rasmus Havmoeller, Lars Johansson, Peter Benedikt Juliusson, Aliasghar A Kiadaliri, Sezer Kisa, Adnan Kisa, Tea Lallukka, Teferi Mekonnen, Tuomo J Meretoja, Atte Meretoja, Mohsen Naghavi, Subas Neupane, Truc Trung Nguyen, Max Petzold, Oleguer Plana-Ripoll, Rahman Shiri, Rannveig Sigurvinsdottir, Vegard Skirbekk, Søren T Skou, Inga Dora Sigfusdottir, Timothy J Steiner, Gerhard Sulo, Thomas Clement Truelsen, Tommi Juhani Vasankari, Elisabete Weiderpass, Stein Emil Vollset, Theo Vos, Simon Øverland

## Abstract

**Background:**

The Nordic countries have commonalities in gender equality, economy, welfare, and health care, but differ in culture and lifestyle, which might create country-wise health differences. This study compared life expectancy, disease burden, and risk factors in the Nordic region.

**Methods:**

Life expectancy in years and age-standardised rates of overall, cause-specific, and risk factor-specific estimates of disability-adjusted life-years (DALYs) were analysed in the Global Burden of Diseases, Injuries, and Risk Factors Study (GBD) 2017. Data were extracted for Denmark, Finland, Iceland, Norway, and Sweden (ie, the Nordic countries), and Greenland, an autonomous area of Denmark. Estimates were compared with global, high-income region, and Nordic regional estimates, including Greenland.

**Findings:**

All Nordic countries exceeded the global life expectancy; in 2017, the highest life expectancy was in Iceland among females (85·9 years [95% uncertainty interval [UI] 85·5–86·4] *vs* 75·6 years [75·3–75·9] globally) and Sweden among males (80·8 years [80·2–81·4] *vs* 70·5 years [70·1–70·8] globally). Females (82·7 years [81·9–83·4]) and males (78·8 years [78·1–79·5]) in Denmark and males in Finland (78·6 years [77·8–79·2]) had lower life expectancy than in the other Nordic countries. The lowest life expectancy in the Nordic region was in Greenland (females 77·2 years [76·2–78·0], males 70·8 years [70·3–71·4]). Overall disease burden was lower in the Nordic countries than globally, with the lowest age-standardised DALY rates among Swedish males (18 555·7 DALYs [95% UI 15 968·6–21 426·8] per 100 000 population *vs* 35 834·3 DALYs [33 218·2–38 740·7] globally) and Icelandic females (16 074·1 DALYs [13 216·4–19 240·8] *vs* 29 934·6 DALYs [26 981·9–33 211·2] globally). Greenland had substantially higher DALY rates (26 666·6 DALYs [23 478·4–30 218·8] among females, 33 101·3 DALYs [30 182·3–36 218·6] among males) than the Nordic countries. Country variation was primarily due to differences in causes that largely contributed to DALYs through mortality, such as ischaemic heart disease. These causes dominated male disease burden, whereas non-fatal causes such as low back pain were important for female disease burden. Smoking and metabolic risk factors were high-ranking risk factors across all countries. DALYs attributable to alcohol use and smoking were particularly high among the Danes, as was alcohol use among Finnish males.

**Interpretation:**

Risk factor differences might drive differences in life expectancy and disease burden that merit attention also in high-income settings such as the Nordic countries. Special attention should be given to the high disease burden in Greenland.

**Funding:**

Bill & Melinda Gates Foundation. The work on this paper was supported by the Research Council of Norway through FRIPRO (project number 262030) and by the Norwegian Institute of Public Health.

## Introduction

The Nordic region consists of the countries Denmark, Finland, Iceland, Norway, and Sweden, and three smaller autonomous areas of two of the countries: Greenland and Faroe Islands (Denmark), and Åland Islands (Finland). Altogether, 27·1 million people inhabit the region (appendix p 2). The countries have a strong tradition of collaboration, and the shared features of policy and welfare systems in the region are referred to as the Nordic model. Social security for inhabitants is the core of the Nordic model, which includes free higher education, a social safety net for people with reduced health and work capacities, and a universal and predominantly publicly financed health-care system. In general, the Nordic countries rank highly on a range of sociodemographic and health-relevant measures, such as the UN Human Development Indices and Indicators, gender equality, educational attainment, and labour force participation. Furthermore, while they are among the countries in the world with the highest gross domestic product (GDP) per capita, they are also ranked among the countries with lowest income disparities within the Organisation for Economic Co-operation and Development area. However, there are also notable diversities between the countries in terms of history, societal development, immigration, cultures, and lifestyles. These differences can cause variation in disease burden.

**Research in context**

**Evidence before this study**

The Nordic countries—Norway, Sweden, Finland, Iceland, and Denmark—rank highly on life expectancy and development indices. Previous comparative studies of the Nordic countries have observed differences in middle-age mortality, cardiovascular diseases, and lung cancer and variations in smoking prevalence, alcohol consumption, and alcohol-related mortality between these countries. Despite a relatively rich health data environment in these countries, broad analyses of differences in life expectancy and disease burden between them have not been done. The Global Burden of Diseases, Injuries, and Risk Factors Study (GBD) has described the contribution of fatal and non-fatal causes and risk factors to disease burden in different geographical locations, including the Nordic countries. However, the Nordic results have been part of the overall presentation of results in GBD, and studies focusing specifically on life expectancy and disease burden in the Nordic countries have been lacking.

**Added value of this study**

The present study expands on previous single studies in the Nordic countries by including non-fatal diseases and a range of risk factors, comparing the disease burden impact of 167 causes and 39 risk factors using the GBD framework. The results are based on data from nearly 4000 sources in the Nordic countries, while also incorporating data from elsewhere if Nordic data are sparse or absent, ensuring disease burden estimates for all causes and risk factors. The standardised data management and methods employed in GBD ensure comparability of results between diseases and risk factors, and across countries and over time. We used the country-specific estimates of GBD 2017 to compare life expectancy and disability-adjusted life-years (DALYs) and the risk factors driving the size of these metrics for the Nordic countries. Greenland, an autonomous area of Denmark, was analysed as a separate location in the GBD study and included in the comparison. Sex-specific analyses cover the period from 1990 to 2017. The complete overview over the population health in the Nordic countries provided by the present study, and the evidence of differences and similarities in life expectancy and disease burden between them, adds valuable information to the details known from previous studies.

**Implications of all the available evidence**

The Nordic countries have the potential to build on their existing strong tradition of collaboration to face both shared and country-specific public health challenges. The high disease burden due to smoking and alcohol use in Denmark, and alcohol use in Finland, implies potential health gains by adopting public health strategies from Iceland, Norway, and Sweden. The low life expectancy and high disease burden in Greenland compared with the rest of the region are striking and require action.

The health situation in the Åland Islands and Faroe Islands is considered comparable to that of Finland and Denmark.^1^ Greenland, however, has large challenges related to adverse childhood experiences, poor mental health, obesity, substance misuse and dependence, smoking, and suicide.^2^ These challenges are particularly present in the Inuit population,^2^ which constitute around 90% of the population.

The Nordic region is known for its relatively rich and comparable health data environment; however, broad analyses of differences in disease burden between the countries are lacking. The aim of the present study was to compare life expectancy and disease burden between countries and sexes in the Nordic region. Based on data from the Global Burden of Disease Study 2017 (GBD 2017), we explored changes in life expectancy from 1990 to 2017, and the top ten causes and risk factors for disability-adjusted life-years (DALYs) among females and males in the Nordic countries and Greenland.

## Methods

### Overview

GBD analyses adhere to the Guidelines for Accurate and Transparent Health Estimates Reporting standards.^3^ Detailed descriptions of measures and methods employed in the GBD study have been previously published.^4^

GBD has produced disease burden estimates by country since GBD 2010, with annual updates since GBD 2015. Each cycle includes new data sources and methodological advancements, and re-analyses the entire time series of results. In GBD 2017, disease burden was estimated for 282 causes of death, 359 diseases and injuries, and 84 risk factors for 195 countries and territories by sex, age, and year. Causes and risk factors are structured into four-level classification hierarchies, increasing in detail from Level 1 to Level 4 (appendix p 2). On each level, the causes and risk factors are exhaustive and mutually exclusive. In this study, the 167 causes and 39 risks at Level 3 in the hierarchy were examined.

GBD employs four main measures of disease burden: deaths, years of life lost (YLLs), years lived with disability (YLDs), and disability-adjusted life-years (DALYs; panel). Additionally, the present study includes measures of life expectancy.

Panel **Measures of disease burden in the Global Burden of Diseases, Injuries, and Risk Factors Study included in the current study**

**Life expectancy**

Life expectancy at birth is the number of years a person in a given location can expect to live, given the observed age-specific mortality rates remain constant.

**YLL**

The YLL is a measure of mortality, quantified as the remaining life expectancy at age of death for a specific cause in a given location. For this measure, life expectancy is defined as the maximum attainable life expectancy, based on a standard life table of the lowest observed death rates in 5-year intervals in populations above 5 million. The same reference is used for both males and females.

**YLD**

The YLD is a measure of non-fatal health loss. It is estimated by multiplying prevalence of a disease or injury with the associated health loss, quantified by disability weights.

**DALY**

The DALY measure indicates the difference between the current health state of a population and an ideal situation where everyone lives to maximum attainable life expectancy in full health. DALYs are the sum of mortality (YLLs) and non-fatal health loss (YLDs) in the population.

YLL=year of life lost. YLD=year lived with disability. DALY=disability-adjusted life-year.

### Data sources

An aim in GBD is to identify and use all available health data sources in the analyses. Information on cause prevalence and incidence is gathered through systematic searches and reviews of published and unpublished data. Information about the input data sources can be found in the GBD Global Health Data Exchange (GHDx) platform. Data on mortality and causes of death are, for the Nordic countries, collected from vital registration systems, such as cause of death registries. Deaths with unspecified diagnoses or codes that cannot be underlying causes of death—so-called garbage codes, such as heart failure—are redistributed in GBD to valid death codes (eg, for heart failure, to coronary heart disease, atrial fibrillation, and other causes) according to algorithms. The proportion of redistributed garbage codes toward the end of the present study period (2015–16) varied from 34% in Greenland to 4% in Finland, with 15–16% in Denmark, Norway, and Sweden.^5^

### Disease burden estimation

Cause of Death Ensemble modelling (CODEm) is used in GBD to estimate causes of death by age, sex, geography, and time.^6^ Estimates of incidence, prevalence, excess mortality, and remission are calculated in DisMod-MR 2.1, a Bayesian meta-regression tool.^7^ Assumed independent comorbidity is factored into the estimates. Disability weights quantify health loss associated with non-fatal causes. They range from 0 (“no health loss”) to 1 (“dead”) and are based on the general population's consideration of cause-specific health loss.^8^

The attributable disease burden from risk factors is estimated in three steps. First, meta-analyses of the published literature are done to estimate the relative risk of non-fatal health loss, mortality, or both, for each risk factor–outcome pair. Next, the current distribution of exposure to risk factors is estimated by location, sex, and age group. Finally, attributable disease burden is estimated by comparing the burden due to the current risk factor distribution with the hypothetical burden due to the theoretical minimum risk exposure level distribution.

### Uncertainty analysis

The uncertainty intervals (UIs) of the point estimates reflect uncertainty from model specification, stochastic variation, and measurement bias. The final published GBD estimates and UIs are based on 1000 draws from the posterior distribution of estimates.^9^ The mean of the draws defines the point estimate, and the 2·5th and 97·5th percentiles define the bounds of the 95% UIs.

### Analysis presented in this paper

We obtained data from the GBD results tool. Location-specific estimates were produced in GBD for the Nordic countries (Denmark, Finland, Iceland, Norway, and Sweden) and the Danish autonomous area of Greenland. The Faroe and Åland Islands were included in the Danish and Finnish estimates. We examined sex-location-specific development in life expectancy by year and percentage change between 1990 and 2017 for the Nordic countries and Greenland, and compared these with global estimates and estimates for the GBD high-income super-region (ie, western Europe, southern Latin America, high-income countries in North America and Asia Pacific, and Australasia) and the Nordic region. The Nordic regional estimates are the overall estimates for the Nordic countries and for Greenland. We identified and compared the ten Level 3 causes contributing the highest age-standardised rates of YLLs, YLDs, and DALYs per 100 000 population by sex, and the ten most important risk factors for DALYs. Location-specific point estimates outside the UIs of the Nordic regional estimates were interpreted as different from the regional estimate. We calculated the proportional difference between the location-specific and the Nordic regional point estimates for the top ten causes and risk factors. Finally, we calculated the percentage of the total number of DALYs attributable to the risk factors included in GBD.

### Role of the funding source

The funders had no role in study design, data collection, data analysis, data interpretation, or report writing. All authors had full access to all data. The corresponding author had final responsibility to submit the manuscript for publication.

## Results

**Figure 1:**
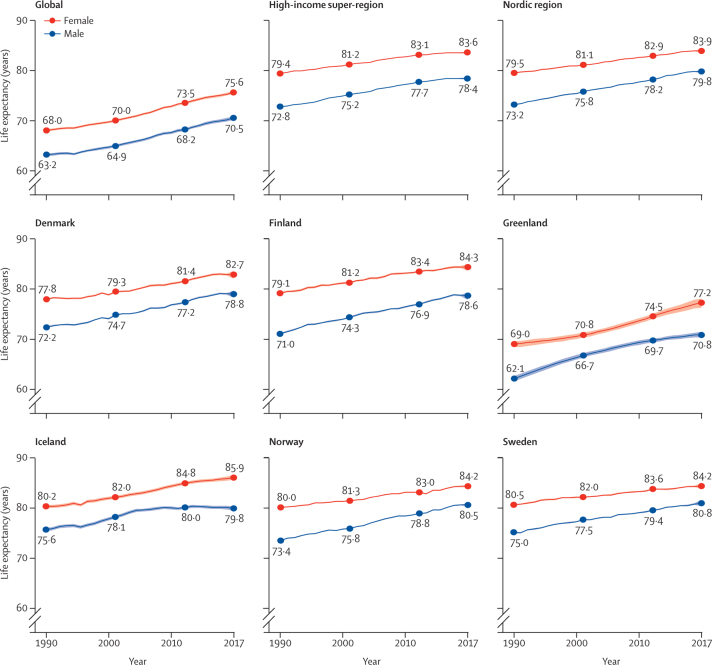
Life expectancy at birth by sex in the years 1990, 2000, 2010, and 2017 The shaded areas around the lines indicate 95% uncertainty intervals.

Life expectancy at birth increased for both sexes in all areas of the Nordic region between 1990 and 2017 (figure 1; appendix p 3). Over the period, males in the Nordic region had 0·5–0·6 years longer life expectancy than males in the high-income region, whereas life expectancy among Nordic females was similar to those in the high-income region. In 1990, males in the Nordic region had 10·0 years longer life expectancy than the global estimate and females had 11·5 years longer life expectancy. This gap reduced to 9·3 years for males and 8·3 years for females in 2017.

The difference in male life expectancy among Nordic countries reduced between 1990 and 2017 (figure 1; appendix p 3). In 1990, the gap between the highest (Iceland) and lowest (Finland) life expectancies was 4·6 years. This dropped to 2·2 years in 2017 (Sweden *vs* Finland). Between-country differences increased slightly among females over the same period, from 2·7 years between Sweden and Denmark in 1990 to 3·2 years between Iceland and Denmark in 2017. Among Nordic countries, Finnish males and Icelandic females improved their life expectancy the most during the studied period (appendix p 3). In the autonomous area of Greenland, life expectancy increased by 8·2 years for females and 8·7 years for males between 1990 and 2017, representing the highest increase in the Nordic region overall. In the Nordic countries, the lowest country-specific life expectancies in 2017 were found for males in Denmark (78·8 years, 95% UI 78·1–79·5) and Finland (78·6 years, 77·8–79·2) and females in Denmark (82·7 years, 81·9–83·4). Greenland had shorter life expectancy than Denmark in 2017, with a gap of 8·0 years for males and 5·5 years for females. Life expectancy in Greenland was more similar to the global estimates than to the Nordic or high-income estimates across the years studied. Sex differences generally reduced over the period; however, a stagnation in life expectancy among males from 2010 to 2017 led to an increased sex difference in Iceland (figure 1). Finland had the largest sex difference across the period, calculated as the average difference for each country across the GBD years studied.

In 2017, Nordic males had 44% fewer DALYs than males globally, whereas females had 41% fewer than females globally in 2017 (table). YLLs also contributed less to the total number of DALYs in the Nordic region than in the high-income region and globally. However, there were sex differences: more than half (53·9%) of the total number of DALYs among males in the Nordic region were due to YLLs, compared with 43·4% among females. All-cause DALY rates did not differ much between the Nordic countries; however, they were substantially higher among males and females in Greenland (table). Overall, all-cause DALY rates were higher among males than among females. The top ten causes of YLLs and YLDs are presented in the appendix (pp 4–7). Age-standardised rates with 95% UIs for DALYs are given in the appendix (pp 8–9).

**Table:** Age-standardised rates of all-cause DALYs, with percentage of total DALYs due to YLLs

		**Females**		**Males**	
		All-cause age-standardised DALY rate (95% UI)	Percentage of total DALYs due to YLLs	All-cause age-standardised DALY rate (95% UI)	Percentage of total DALYs due to YLLs
Global		29 934·6 (26 981·9–33 211·2)	60·4%	35 834·3 (33 218·2–38 740·7)	70·5%
High income		18 678·8 (15 790·6–22 022·9)	44·4%	22 044·2 (19 487·1–24 983·3)	57·2%
Nordic region		17 762·2 (14 873·2–21 228·4)	43·4%	19 932·7 (17 281·4–22 954·3)	53·9%
	Denmark	18 330·9 (15 503·9–21 650·3)	46·7%	20 696·0 (17 964·2–23 821·3)	56·3%
	Finland	17 524·3 (14 522·1–20 886·8)	43·6%	21 776·2 (19 010·9–24 970·2)	57·0%
	Greenland	26 666·6 (23 478·4–30 218·8)	52·6%	33 101·4 (30 182·3–36 218·6)	68·8%
	Iceland	16 074·1 (13 216·4–19 240·8)	35·5%	19 417·3 (16 828·9–22 390·5)	52·1%
	Norway	17 794·2 (14 811·1–21 288·1)	40·4%	19 646·9 (16 916·5–22 733·0)	49·4%
	Sweden	17 563·1 (14 584·4–20 977·3)	42·9%	18 555·7 (15 968·6–21 426·8)	52·5%

DALY=disability-adjusted life-year. UI=uncertainty interval. YLL=year of life lost.

**Figure 2:**
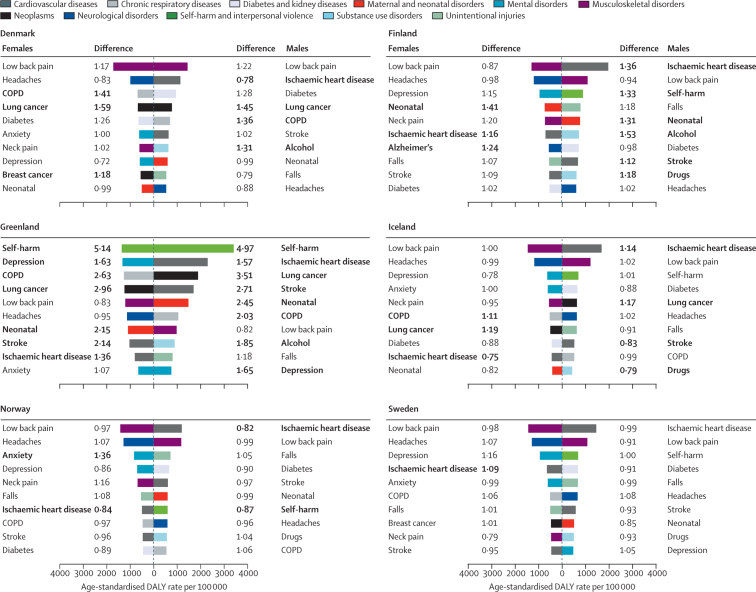
Age-standardised DALY rates per 100 000 by sex for the top ten Level 3 causes in the Nordic countries in 2017, and difference from the Nordic region estimate Difference is expressed as proportional difference—eg, a difference of 1·17 indicates that the rate is 17% higher compared with the Nordic region estimate. Bold differences indicate that the country-specific point estimate is outside the 95% uncertainty interval of the Nordic region estimate. Anxiety=anxiety disorders. Alcohol=alcohol use disorders. Alzheimer's=Alzheimer's disease and other dementias. COPD=chronic obstructive pulmonary disease. Depression=depressive disorders. Drugs=drug use disorders. Headaches=headache disorders. Lung cancer=tracheal, bronchus, and lung cancer. Neonatal=neonatal disorders. DALY=disability-adjusted life-year.

The leading causes of DALYs in the Nordic region differed by sex (figure 2; appendix pp 8–9). Causes that largely contribute to DALYs through YLLs (such as ischaemic heart disease, stroke, lung cancer, diabetes, and chronic obstructive pulmonary disease [COPD]), as well as alcohol and drug use disorders and injuries (ie, falls and self-harm), ranked higher among males than females (appendix pp 4–5). Causes related to YLDs (such as headaches, anxiety disorders, depressive disorders, and low back and neck pain) made up a larger proportion of the DALYs among females (figure 2; appendix pp 6–7). Ischaemic heart disease and low back pain were the leading two causes of DALYs among males, whereas low back pain and headaches were the two leading causes among females.

Most country-specific estimates of DALYs were within the UIs of the Nordic regional estimates (figure 2; appendix pp 8–9). In Greenland, however, higher DALY rates than the regional estimates were found for most of the ten leading causes, with the largest difference found for self-harm. Region–country differences were primarily found for causes of YLLs (appendix pp 4–5). Among the Nordic countries, ischaemic heart disease had high country-specific DALY estimates in Finland, for Swedish females, and Icelandic males, and low estimates in Norway, Denmark, and among Icelandic females (figure 2). Denmark and Iceland had high DALY rates for lung cancer across both sexes. DALY rates due to COPD were high among females in Iceland and both sexes in Denmark. More disease burden was attributed to alcohol use disorders among Danish and Finnish males than their neighbours. Self-harm ranked among the top ten causes of DALYs for males in all countries but Denmark. Self-harm rates were particularly high in Finland and Greenland—for the latter, rates were five times higher than the regional estimates for both males and females (figure 2). In the Nordic countries, among the causes that primarily contribute to YLDs, only anxiety disorders among Norwegian females and neonatal disorders among Finnish males and females differed from the regional estimate (figure 2; appendix pp 6–7).

**Figure 3:**
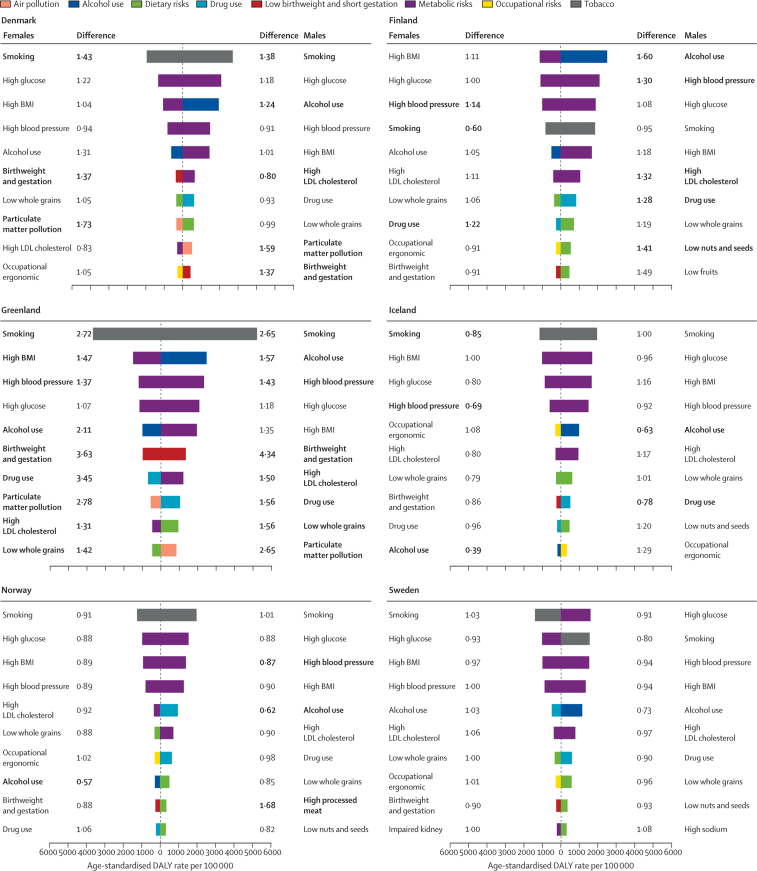
Age-standardised DALY rates per 100 000 by sex for the top ten Level 3 risk factors in the Nordic countries in 2017 and difference from the Nordic region estimate Difference is expressed as proportional difference—eg, a difference of 1·43 indicates that the rate is 43% higher compared with the Nordic region estimate. Bold differences indicate that the country-specific point estimate is outside the 95% uncertainty interval of the Nordic regional estimate. Birthweight and gestation=low birthweight and short gestation. BMI=body-mass index. High blood pressure=high systolic blood pressure. High glucose=high fasting plasma glucose. High processed meat=diet high in processed meat. Impaired kidney=impaired kidney function. Low fruits=diet low in fruits. Low nuts and seeds=diet low in nuts and seeds. Low whole grains=diet low in whole grains. Occupational ergonomic=occupational ergonomic factors. DALY=disability-adjusted life-year.

Among the Nordic countries, the proportion of DALYs attributable to the GBD risk factors in 2017 was higher in males (country range 41·4–49·0%) than females (31·6–39·6%; appendix pp 10–11). There were few differences in the top ten ranking of risk factors (figure 3; appendix pp 12–13). Males and females in Greenland had higher rates than the regional estimates for most of these risk factors. Smoking ranked as the top risk factor for both males and females in all countries, except for males in Sweden (second) and for both sexes in Finland (fourth). Males and females in Denmark had around 40% more DAYs due to smoking than the regional estimate, and Greenland had almost three times more than the regional estimate. Alcohol use was among the top ten risk factors for both sexes in all countries, as was drug use except for Danish and Swedish females. Among the Nordic countries, DALYs attributable to alcohol use were particularly high among males in Finland and Denmark. The metabolic risk factors (ie, high fasting plasma glucose, high systolic blood pressure, high body-mass index, and high LDL cholesterol) ranked among the top six risk factors in all the Nordic countries and for both sexes, except for high LDL cholesterol, which ranked eighth among Danish females. Most country estimates for these risk factors differed little from the Nordic regional estimates; exceptions were high systolic blood pressure, which was higher among both sexes in Finland and lower among Icelandic females and Norwegian males, and high LDL cholesterol, which was higher among Finnish males and lower among Danish males. Dietary factors were generally more important for male than female disease burden (figure 3).

## Discussion

Compared with global estimates, both sexes had higher life expectancy and lower overall disease burden in terms of age-standardised DALYs in the Nordic region. Premature mortality (YLLs) was less important for the overall disease burden in the Nordic region than globally, and among Nordic females compared with males. The overall disease burden did not differ substantially between the Nordic countries, but some differences were found for life expectancy, which was lower for both sexes in Denmark and for males in Finland. Most of the top ten causes of disease burden among males were related to mortality (YLLs), whereas causes related to disability (YLDs) were ranked higher as causes of disease burden among females. Sex-specific country variation was mainly observed for causes contributing to disease burden primarily through YLLs. Alcohol-attributable disease burden was higher among males in Denmark and Finland than in the other Nordic countries, as was disease burden attributable to smoking in Denmark. Males and females in Greenland had substantially lower life expectancy and higher all-cause DALY rates than the Nordic countries.

The country variations identified in our study were in line with previous studies focusing on differences between the Nordic countries in smoking prevalence^10^ and smoking-related mortality,^11^ alcohol consumption^12^ and alcohol-related mortality,^11^, ^13^, ^14^ middle-aged mortality,^15^ and cause-specific mortality.^15^ By comparing 167 Level 3 causes and 39 Level 3 risk factors in a single analytic framework, the GBD results expand the knowledge from previous single studies. The inclusion of sex-specific and country-specific drivers for non-fatal diseases is important for benchmarking and comparative analyses in the Nordic region. The results suggest that the individual countries face both common and specific challenges in reducing disease burden.

Life expectancy became more similar between the Nordic countries from 1990 to 2017. The general increase was stronger in the first half of this period than the second half. This trend is in line with findings from other countries and periods; rapid progress in some periods is often followed by a slower rise.^16^ After World War 2, and particularly after the 1970s, the Nordic countries had a faster growth in welfare, living standards, and health care than most other countries in the world. For long periods in the post-war era, Iceland, Sweden, and Norway had the highest life expectancy in global comparisons. However, other countries in other regions have caught up with the Nordic countries in economic, social, and cultural modernisation, and are now in many cases exceeding the Nordic countries in life expectancy ranking. A complex interplay of factors could explain why the Nordic countries have had a relative decline in life expectancy rank. One factor might be that other countries have had more success in improving mortality in older ages.^17^ As the vast majority of deaths in the Nordic countries happens after age 65 years, future studies should compare the development in causes related to death in older age to identify whether the Nordic countries are less successful in reducing these than countries that have surpassed the life expectancies in Nordic countries.

Another factor for why the Nordic countries are losing ground in life expectancy rankings might relate to what is often termed the Nordic paradox. This term refers to the observation that despite the low income inequalities, free access to education and health services, and generous welfare policies that characterise Nordic countries, gaps in life expectancy by socioeconomic status are larger in the Nordic welfare states than in many less egalitarian societies.^18^ Furthermore, this gap seems to have increased over the past few decades.^19^ This suggests that at least part of the slowing in improvement of life expectancy in the Nordic countries could be due to a stagnation in life expectancy among those with low socioeconomic status. This should be a common cause for concern, which could be addressed collectively and in joint effort by all Nordic countries.

Cardiovascular diseases and cancers were important causes of DALYs in all countries studied, illustrating the need for a continued focus on prevention and treatment of these diseases. Smoking is responsible for an estimated 16·4% of the cardiovascular burden and 23·7% of the cancer burden in the Nordic region (data not shown). Smoking-attributable disease burden did, however, vary between countries, with the Danes, and in particular Danish females, experiencing a much higher disease burden due to smoking than the other countries. It is likely that these country differences are related to differences in smoking policies and culture. For instance, Norway has had strict tobacco legislation for decades. The widespread and longstanding use of snus as a tobacco alternative in Sweden might have contributed to the lower smoking-related disease burden in this country, as snus is not captured within GBD tobacco use estimates.^20^ By comparison, Denmark has a shorter history of tobacco control^21^ and might therefore have a larger potential to reduce its smoking-related disease burden by adopting its Nordic neighbours' policies. Denmark, Norway, Iceland, and Sweden were among the 13 countries globally experiencing the largest declines in smoking prevalence between 1990 and 2015.^22^ Smoking is also much less common in younger generations,^23^ although a high level of smoking across the region among young women in lower socioeconomic positions is a challenge. Despite this, a high smoking-attributable disease burden in the Nordic region is still to be expected due to population growth and ageing. However, if the Nordic countries continue their success in smoking cessation and prevention of young people starting smoking, the disease burden attributed to smoking could reduce in the coming decades.

Alcohol use ranked highly among risk factors. It was the leading risk factor among males in Finland and ranked third among Danish males. Overall alcohol consumption levels in these two countries are similar,^24^ but consumption of stronger alcohol is higher in Finland than in Denmark.^24^ Sex differences in DALYs attributed to alcohol use were also larger in Finland than in Denmark. These results are in line with previous GBD publications.^25^ Alcohol policies differ somewhat between the Nordic countries, which might contribute to differences in alcohol consumption and disease burden. Iceland, Sweden, Norway, and Finland have historically had restrictive alcohol policies, mainly through strict regulation of alcohol sales, advertisement, age limits for purchasing, pricing, and taxation.^24^ In the mid-1990s, the Nordic countries had to adapt their alcohol polices to the framework of the EU and the European Economic Area. Being outside of the EU, Iceland and Norway were able to maintain a stricter alcohol policy than the other Nordic countries. Alcohol policies were liberalised in Finland, Sweden, and Denmark, and in 2004, alcohol prices were also reduced in Finland. Geography might also influence access to these substances, and imported alcohol for personal use can vary by proximity to and trade regulation with countries with more affordable alcohol products. For instance, traveller quotas for alcohol imports from Estonia, bordering Finland, were abolished when Estonia joined the EU in 2004. Together with the reduction of alcohol prices seen the same year, this led to an increase in consumption and mortality rates from alcohol-related causes such as liver cirrhosis and injuries in Finland.^26^ Nevertheless, men and women in the same country share policy context. The observed sex differences in alcohol-attributable disease burden within the Nordic countries, and in Finland particularly, implies that cultural, social, and gender-specific factors are also involved, adding to the ongoing debate on the effects of alcohol policies.^11^

In terms of dietary risk, the Nordic countries face many of the same challenges as seen globally.^27^ A healthy diet is not only about avoiding what is deemed bad, but also replacing this with intake of what is deemed good. In 2006, the Nordic Council of Ministers launched an action plan for heathier diets in the Nordic countries. Many of the interventions suggested in this plan targeted consumers. However, current expert recommendations for improving population diet point further upstream, targeting the food industry and systems for food access.^27^ Country-specific interventions in the Nordic region include access to nutritional food in institutions such as schools and hospitals, food product labelling, pricing, and taxation, as well as collaboration with the food industry in terms of the composition of ingredients in its products. A renewed comparative focus on these interventions could help the Nordic countries to learn from each other to improve the population's diet.

Headache, musculoskeletal disorders, and mental disorders were important causes of non-fatal disease burden across the countries, and entail major economic consequences for society through work absence and health-service use. Back pain, neck pain, anxiety disorders, and depressive disorders are large contributors to disease burden, particularly in the working-age population (aged 15–67 years), despite no mortality attributed to these causes. Compared with causes with a large mortality component, effective prevention of disease burden by reducing causes of disability is less well established. This is mirrored in GBD, because only fractions of the disease burden from these causes are assigned risk factors; for example, the risk factors included in GBD explain 94·9% of the DALYs attributed to ischaemic heart disease. In GBD 2017, bullying victimisation was introduced as a risk factor for anxiety and depressive disorders, accounting for 1·7% and 3·1%, respectively, of their disease burden in the Nordic region. Implementation and improvement of anti-bullying interventions is thus one identified potential strategy for reducing disease burden from mental disorders. Beyond better identification of modifiable risk factors for prevention, tools to reduce severity levels and the personal, social, and economic consequences associated with important non-fatal causes of disease burden seem warranted.

Denmark has specific public health challenges compared with the other Nordic countries. A range of publications have pointed at probable causes of the poorer population health in Denmark, and higher rates of smoking and alcohol use have been particularly emphasised. Despite the alarming estimates of alcohol and tobacco use in Denmark, access to these products remains liberal. For instance, a WHO report^21^ in 2018 noted that Denmark had no overall strategy or plan to protect children, adolescents, and adults from the harms of tobacco; that tobacco was both affordable and promoted at sales points; and that the tobacco industry was influential in Danish policy making. The experience from Denmark indicates that information alone might not be sufficient to markedly change health behaviour, and that targeted political efforts is often necessary to be able to change risk factor and disease burden patterns in the population.

The disease burden profile in Greenland stands in sharp contrast to that of the Nordic countries. Bjerregaard and Larsen^2^ have highlighted three main determinants of the problematic health conditions in Greenland: adverse childhood conditions, obesity, and smoking. Additionally, the GBD data highlight alcohol and drug use and metabolic factors. Greenland has poorer socioeconomic conditions than the Nordic countries, and faces a social transition with important changes in lifestyle and employment. Additionally, the traditional fishing and hunting lifestyle implies a higher risk of accidents, and availability of guns might contribute to high rates of suicide and homicide by firearm (data not shown). Similar problems have been described from other Inuit populations—eg, in Canada.^28^

The key strength of GBD is the standardised data management and methods to make results comparable. In terms of limitations, this study shares those of GBD more generally, which are described in the capstone papers. The key limitation for the present purpose is the large spread in the quality of the underlying data between estimates of life expectancy and causes of death, and estimates of non-fatal and risk factor-attributed disease burden. GBD provides results for all included risks, diseases, and injuries even when data are sparse. This ensures estimates for each cause and risk factor, but requires caution when interpreting and using the results. For non-fatal causes of disease burden, underlying data sources are sparser and more heterogeneous than mortality sources, and comparisons of non-fatal estimates between countries are further hampered by the lack of information on severity of disease, which makes it difficult to account for differences in quality and access to treatment. Despite these shortcomings, GBD provides the by far most comprehensive and reliable framework for comparisons of non-fatal health outcomes between countries. For mortality data, the Nordic countries have high-quality, continuously updated cause of death registries. GBD data are similar to the national statistics for life expectancy and overall mortality estimates. Thus, the use of GBD data for these measures might not represent any particular advantage from the official, national statistics of the Nordic countries. For cause of death data, however, there are several advantages of using model-based GBD data instead of direct comparisons of national statistics, as the GBD models account for idiosyncratic and system-adapted differences in national data over time and between countries, and ensure that all garbage codes are redistributed in a standardised way. This ensures that the GBD results are internally comparable,^5^ but comparisons with national mortality statistics for causes where garbage code redistribution is extensive will reveal discrepancies. One such example is Greenland, which had a higher proportion of garbage codes than the Nordic countries. Redistribution of garbage codes in Greenland will not affect life expectancy and all-cause mortality estimates in this location, but can affect the resulting distribution of causes of death.

Despite the need for better and more updated data on non-fatal diseases and risk factors, the availability of high-quality data is better in the Nordic countries than in many other regions in the world. As data sources are generally similarly organised in the Nordic countries, cross-country comparisons should be reasonably reliable in the Nordic region. Comparisons between GBD estimates and national vital statistics from the Nordic countries might also help to adjust and improve the GBD models, which will also be useful for improving estimates from regions of the world with poorer data.

The life expectancy and disease burden in the Nordic region share common patterns, but with several important differences between the countries and the sexes. Greenland has a disease burden profile in sharp contrast to the Nordic countries. Comparisons between the Nordic countries illustrate how disease burden can vary despite similar geographical and income settings and comparable access to universal health and welfare services, but with cultural and lifestyle differences. The observations suggest a potential for reduced disease burden through adopting evidence-based policies and programmes from neighbouring countries with lower disease burden.
