# Supplementary material for: Life expectancy and disease burden in the Nordic countries: results from the Global Burden of Diseases, Injuries, and Risk Factors Study 2017
Source: Lancet Public Health. 2019 Nov 20;4(12):e658–69. doi: 10.1016/S2468-2667(19)30224-5 (PMC7098475; doi:10.1016/S2468-2667(19)30224-5)
Supplement: Supplementary appendix [file mmc1.pdf]

THE LANCET
Public Health

Supplementary appendix

This appendix formed part of the original submission and has been peer reviewed. We post it as supplied by the authors.

Supplement to: Nordic Burden of Disease Collaborators. Life expectancy and disease burden in the Nordic countries: results from the Global Burden of Diseases, Injuries, and Risk Factors Study 2017. *Lancet Public Health* 2019; published online Nov 20. https://doi.org/10.1016/S2468-2667(19)30224-5.

Contents

Appendix table 1. Population in the Nordic region in 2017. 2

Appendix table 2. Hierarchical organisation of causes and risk factors in the GBD study. 2

Appendix table 3. Development in life expectancy (LE) at birth in the Nordic countries from 1990 to 2017 by years and percent, males and females..... 3

Appendix table 4a. Age-standardised Years of Life Lost (YLL) rates per 100,000 population of top-ten causes (Level 3) in the Nordic countries in 2017, males. 4

Appendix table 4b. Age-standardised Years of Life Lost (YLL) rates per 100 000 population of top-ten causes (Level 3) in the Nordic countries in 2017, females. 5

Appendix table 5a. Age-standardised Years Lived with Disability (YLD) rates per 100,000 population of top-ten causes (Level 3) in the Nordic countries in 2017, males. 6

Appendix table 5b. Age-standardised Years Lived with Disability (YLD) rates per 100,000 population of top-ten causes (Level 3) in the Nordic countries in 2017, females. 7

Appendix table 6a. Age-standardised Disability-Adjusted Life-Years (DALY) rates per 100,000 population of top-ten causes (Level 3) in the Nordic countries in 2017, males. 8

Appendix table 6b. Age-standardised Disability-Adjusted Life-Years (DALY) rates per 100,000 population of top-ten causes (Level 3) in the Nordic countries in 2017, females. 9

Appendix table 7a. Number of disability-adjusted life-years (DALY) and percent of total DALYs attributed to top-ten risk factors1 in the Nordic countries, males. 10

Appendix table 7b Number of disability-adjusted life-years (DALY) and percent of total DALYs attributed to top-ten risk factors1 in the Nordic countries, females. 11

Appendix table 8a. Age-standardised disability-adjusted life-years (DALY) rates per 100,000 population of top-ten risk factors (Level 3) in the Nordic countries in 2017, males. 12

Appendix table 8b. Age-standardised disability-adjusted life-years (DALY) rates per 100,000 population of top-ten risk factors (Level 3) in the Nordic countries in 2017, females. 13

Appendix table 1. Population in the Nordic region in 2017.

Country/location	Population
Iceland	338,349
Norway	5,258,317
Sweden	9,995,153
Finland	5,503,297
Åland[1]	29,214
Denmark	5,748,769
Faroe Islands[2]	49,864
Greenland[2]	55,860

[1] Autonomous area of Finland, [2] Autonomous area of Denmark

Appendix table 2. Hierarchical organisation of causes and risk factors in the GBD study.

Levels	Causes	Risk factors
Level 0	All causes combined	All risk factors combined
Level 1	Three broad groups: i) communicable, maternal, neonatal, and nutritional diseases (CMNN); ii) non-communicable diseases (NCDs); and iii) injuries	Three broad groups: i) environmental and occupational risk factors; ii) metabolic risk factors, iii) behavioural risk factors
Level 2	22 causes	19 risks
Level 3	167 causes	39 risks
Level 4	288 causes	22 risks
Sequelaes	3484	
Impairments	9	
Nature of injury	7 aggregates	

Appendix table 3. Development in life expectancy (LE) at birth in the Nordic countries from 1990 to 2017 by years and percent, males and females.

Males	LE 1990 (95% UI)	LE 2017 (95% UI)	Dev. Years	Dev. %
Global	63.2 (62.9-63.4)	70.5 (70.1-70.8)	7.3	11.6 %
High-income region	72.8 (72.7-72.8)	78.4 (78.2-78.6)	5.7	7.8 %
Nordic Region	73.2 (73.1-73.3)	79.8 (79.5-80.1)	6.6	9.0 %
Denmark	72.2 (72.1-72.2)	78.8 (78.1-79.5)	6.6	9.1 %
Finland	71.0 (70.9-71.2)	78.6 (77.8-79.2)	7.5	10.6 %
Greenland	62.1 (61,6-62.6)	70.8 (70.3-71.4)	8.8	14.1 %
Iceland	75.6 (75.2-75.9)	79.8 (79.4-80.2)	4.3	5.7 %
Norway	73.4 (73.3-73.5)	80.5 (80.2-80.7)	7.1	9.6 %
Sweden	75.0 (74.8-75.1)	80.8 (80.2-81.4)	5.8	7.8 %
**Females**	**LE 1990 (95% UI)**	**LE 2017 (95% UI)**	**Dev. Years**	**Dev. %**
Global	68.0 (67.8-68.3)	75.6 (75.3-75.9)	7.5	11.1 %
High-income region	79.4 (79.4-79.4)	83.6 (83.4-83.7)	4.1	5.2 %
Nordic Region	79.5 (79.4-79.6)	83.9 (83.6-84.2)	4.4	5.5 %
Denmark	77.8 (77.6-77.9)	82.7 (81.9-83.4)	4.9	6.3 %
Finland	79.1 (78.9-79.3)	84.3 (83.6-84.9)	5.2	6.5 %
Greenland	69.0 (68.4-69.5)	77.2 (76.2-78.0)	8.2	11.8 %
Iceland	80.2 (79.8-80.5)	85.9 (85.5-86.4)	5.8	7.2 %
Norway	80.0 (79.9-80.1)	84.2 (84.0-84.4)	4.2	5.2 %
Sweden	80.5 (80.4-80.6)	84.2 (83.7-84.7)	3.7	4.5 %

Abbreviations: LE=life expectancy. Dev. Years=development in years from 1990 to 2017. Dev. %= Development in percent from 1990 to 2017. UI=uncertainty interval

Appendix table 4a. Age-standardised Years of Life Lost (YLL) rates per 100,000 population of top-ten causes (Level 3) in the Nordic countries in 2017, males.

	**Males**													
	**The Nordic region**		**Denmark**		**Finland**		**Greenland**		**Iceland**		**Norway**		**Sweden**	
		**ASR (95% UI)**		**ASR (95% UI)**		**ASR (95% UI)**		**ASR (95% UI)**		**ASR (95% UI)**		**ASR (95% UI)**		**ASR (95% UI)**
**1**	Ischaemic heart disease	1381.4 (1316-1485.6)	Ischaemic heart disease	1077.1 (993.3-1175.8)	Ischaemic heart disease	1895.4 (1736.7-2103.9)	Self-harm	3376.33 (3012.1-3770.8)	Ischaemic heart disease	1600.6 (1519-1695.2)	Ischaemic heart disease	1140.4 (1085.2-1212.3)	Ischaemic heart disease	1356.5 (1260.8-1480.7)
**2**	Self-harm	678.3 (646.3-714.2)	Lung cancer	771.1 (706.7-840.5)	Self-harm	899.8 (821.1-994.3)	Ischaemic heart disease	2254.8 (2073.3-2423.5)	Self-harm	685 (628.5-743.1)	Self-harm	591.3 (557.4-643.6)	Self-harm	681.1 (630.0-740.1)
**3**	Lung cancer	530.6 (507.5-558.5)	Self-harm	517.9 (467.7-570.9)	Cirrhosis	584.6 (520.4-658.2)	Lung cancer	1873.6 (1689.9-2042.3)	Lung cancer	620.8 (575.9-670.2)	Lung cancer	532.2 (506.4-562.1)	Stroke	419.7 (385.4-463.2)
**4**	Stroke	459.4 (434.6-493.8)	Stroke	501.2 (455.9-552.8)	Lung cancer	556.1 (506.5-609.4)	Stroke	1411.5 (1281.5-1528.3)	Stroke	383.2 (353.3-413.8)	Stroke	395.3 (375.6-420.3)	Lung cancer	367.7 (340.4-396.6)
**5**	Alzheimer's disease	317.9 (304.0-330.4)	COPD	492.7 (450.3-539)	Stroke	539.5 (491.3-595.5)	Neonatal disorders	1290.5 (1006.7-1632.9)	Alzheimer's disease	329.6 (312.7-347.6)	Colorectal cancer	362.2 (336.7-388.5)	Prostate cancer	302.8 (217.9-352.5)
**6**	Colorectal cancer	316.6 (299.8-335.6)	Alcohol use disorders	400.0 (354.4-449.4)	Alzheimer's disease	490.5 (460.7-526.3)	COPD	634.8 (551.3-729.6)	Prostate cancer	321.5 (248.5-412.8)	COPD	344.0 (329.5-364.1)	Drug use disorders	302.6 (269.7-338.9)
**7**	Drug use disorders	310.3 (291.5-330.1)	Colorectal cancer	395.4 (355.8-439)	Alcohol use disorders	383.4 (333.6-439.3)	Lower respiratory infections	612.9 (473.7-705.9)	Colorectal cancer	270.2 (246.6-295)	Prostate cancer	343.6 (247.8-419.3)	Colorectal cancer	292.8 (271.5-315.2)
**8**	Prostate cancer	308.5 (223.2-368.5)	Cirrhosis	361.5 (321.0-407.2)	Drug use disorders	361.5 (313.9-408.3)	Alcohol use disorders	555.3 (458.1-777.8)	Lower respiratory infections	260.5 (239.9-284.4)	Drug use disorders	323.0 (297.2-343.7)	Alzheimer's disease	262.0 (247.1-279)
**9**	Cirrhosis	296.8 (272.3-320.1)	Neonatal disorders	353.9 (260.6-467.6)	Road injuries	284.6 (255.6-316.6)	Colorectal cancer	519.9 (464.9-572.5)	Road injuries	254.0 (228.3-283.3)	Alzheimer's disease	296.9 (277.7-316.3)	Neonatal disorders	240.1 (181.6-296.3)
**10**	COPD	295.9 (277.8-318.4)	Prostate cancer	347.9 (233.7-400.3)	Falls	271.1 (246.1-300.5)	Drowning	450.5 (386.5-516.9)	COPD	248.1 (226.4-269.9)	Road injuries	241.0 (230.3-253.2)	Road injuries	217.9 (196.8-239.2)
**Total**	**All causes**	**9627.0 (9309.2-9912.6)**	**All causes**	**10501.1 (9810.1-11238.5)**	**All causes**	**11097.5 (10383.0-11935.6)**	**All causes**	**22642.9 (21348.4-23859.6)**	**All causes**	**9491.7 (9096.3-9918.6)**	**All causes**	**8883.1 (8675.2-9081.0)**	**All causes**	**8615.4 (8098.9-9144.7)**

Abbreviations: ASR=age-standardised rates. COPD=chronic obstructive pulmonary disease. UI=uncertainty intervals

Appendix table 4b. Age-standardised Years of Life Lost (YLL) rates per 100 000 population of top-ten causes (Level 3) in the Nordic countries in 2017, females.

	Females													
	**The Nordic region**		**Denmark**		**Finland**		**Greenland**		**Iceland**		**Norway**		**Sweden**	
		**ASR (95% UI)**		**ASR (95% UI)**		**ASR (95% UI)**		**ASR (95% UI)**		**ASR (95% UI)**		**ASR (95% UI)**		**ASR (95% UI)**
**1**	Ischaemic heart disease	533.4 (506.3-576.5)	Lung cancer	654.7 (585.8-729.8)	Ischaemic heart disease	626.4 (566.6-695.5)	Self-harm	1293.2 (1166.7-1433.7)	Lung cancer	493.0 (449.7-539.8)	Ischaemic heart disease	454.8 (435.3-482.5)	Ischaemic heart disease	570.4 (522.9-628.7)
**2**	Lung cancer	413.0 (392.7-436.3)	Breast cancer	482.9 (436.7-533.5)	Alzheimer's disease	462.3 (428.0-499.4)	Lung cancer	1231.7 (1068.5-1407)	Ischaemic heart disease	404.3 (374.9-435.0)	Lung cancer	392.6 (374.1-413.1)	Breast cancer	401.2 (368.8-436.4)
**3**	Breast cancer	400.8 (380.8-421.5)	COPD	433.6 (390.3-481.9)	Breast cancer	359.6 (323.5-399.4)	Neonatal disorders	897.8 (686.1-1169.1)	Breast cancer	331.2 (302.0-359.6)	Breast cancer	358.2 (339.8-375.8)	Lung cancer	370.3 (339.5-403.7)
**4**	Alzheimer's disease	350.2 (335.8-364.8)	Ischaemic heart disease	427.0 (387.7-471.6)	Stroke	351.9 (317.1-392.4)	Stroke	778.7 (687.6-893.5)	Alzheimer's disease	300.0 (279.3-323.0)	Alzheimer's disease	348.2 (330.6-361.6)	Alzheimer's disease	305.5 (286.6-326.8)
**5**	Stroke	323.8 (305.3-348.8)	Stroke	353.3 (320.6-390.3)	Self-harm	277.5 (249.1-307.0)	Ischaemic heart disease	777.3 (677.7-906.5)	Stroke	213.8 (195.7-233.8)	Stroke	296.4 (281.8-314.5)	Stroke	304.7 (276.8-338.8)
**6**	Self-harm	249.2 (236.2-262.6)	Alzheimer's disease	308.1 (282.4-334)	Lung cancer	257.1 (221.4-297.1)	COPD	758.1 (614.4-892.5)	COPD	193.3 (175.0-212.8)	Colorectal cancer	285.3 (271.3-300.4)	Self-harm	280.0 (254.5-306.4)
**7**	COPD	241.4 (228.2-258.7)	Colorectal cancer	290.3 (260.9-321.9)	Cirrhosis	188.2 (165.7-211.6)	Colorectal cancer	484.2 (416.6-556.6)	Lower respiratory infections	153.9 (140.6-168.6)	COPD	279.1 (266.9-297.2)	Colorectal cancer	233.3 (214.1-253.1)
**8**	Colorectal cancer	238.6 (225.5-252.3)	Neonatal disorders	290.3 (225.0-371.5)	Congenital defects	173.9 (133.0-233.4)	Interpersonal violence	450.7 (325.9-563.4)	Colorectal cancer	145.4 (131.2-159.2)	Self-harm	243.8 (232.9-254.4)	COPD	207.0 (187.2-228.9)
**9**	Neonatal disorders	189.5 (155.5-213.1)	Congenital defects	215.3 (166.5-290.7)	Pancreatic cancer	165.8 (146.5-185.7)	Congenital birth defects	427.4 (320.3-558.7)	Congenital defects	140.6 (94.3-189.1)	Ovarian cancer	159.5 (150.5-168.1)	Congenital defects	189.9 (156.9-287.5)
**10**	Congenital defects	184.6 (153.7-256.9)	Self-harm	173.8 (154.9-193.0)	Colorectal cancer	165.3 (148.8-184.2)	Lower respiratory infections	404.3 (341.6-469.3)	Brain cancer	129.7 (110.6-165.3)	Congenital defects	152.3 (118.9-214.5)	Neonatal disorders	176.1 (133.4-210.9)
**Total**	**All causes**	**6032.4 (5831.4-6244.9)**	**All causes**	**7008.8 (6497.2-7590.3)**	**All causes**	**5780.7 (5324.6-6276.8)**	**All causes**	**14149.8 (13061.9-15375.5)**	**All causes**	**4787.8 (4504.7-5076.6)**	**All causes**	**5776.6 (5634.1-5918.6)**	**All causes**	**5745.9 (5399.8-6110.6)**

Abbreviations: ASR=age-standardised rates. COPD=chronic obstructive pulmonary disease. UI=uncertainty intervals

Appendix table 5a. Age-standardised Years Lived with Disability (YLD) rates per 100,000 population of top-ten causes (Level 3) in the Nordic countries in 2017, males.

	**Males**													
	**The Nordic region**		**Denmark**		**Finland**		**Greenland**		**Iceland**		**Norway**		**Sweden**	
		**ASR (95% UI)**		**ASR (95% UI)**		**ASR (95% UI)**		**ASR (95% UI)**		**ASR (95% UI)**		**ASR (95% UI)**		**ASR (95% UI)**
**1**	Low back pain	1185.8 (843.5-1607.8)	Low back pain	1448.0 (1021.6-1974.9)	Low back pain	1112.0 (790.0-1522.8)	Low back pain	977.2 (695.5-1338.5)	Low back pain	1208.0 (870.7-1607.8)	Low back pain	1176.5 (846.5-1608.9)	Low back pain	1078.9 (769.3-1456.9)
**2**	Headache disorders	614.7 (408.9-861.6)	Diabetes mellitus	689.8 (454.4-972.4)	Neonatal disorders	626.3 (446.6-837.2)	Depressive disorders	752.5 (520.3-1025.8)	Headache disorders	627.9 (414.2-887.3)	Headache disorders	589.7 (393.2-830.6)	Headache disorders	665.4 (442.7-939.5)
**3**	Diabetes mellitus	578.7 (384.3-811.5)	Headache disorders	540.5 (368.1-744.6)	Headache disorders	625.3 (414.1-883.1)	Headache disorders	470.1 (309.6-670.8)	Diabetes mellitus	560.2 (372.6-785.7)	Falls	563.6 (403.5-770.8)	Falls	545.2 (388.3-744.9)
**4**	Falls	522.8 (372.5-716.1)	Neck pain	455.0 (317.4-638.4)	Diabetes mellitus	625.1 (419.7-878.8)	Drug use disorders	430.2 (305.2-567.4)	Falls	460.8 (324.6-637.2)	Neck pain	538.6 (375.2-751.6)	Diabetes mellitus	515.7 (344.9-723.1)
**5**	Depressive disorders	455.3 (322.5-619.5)	Falls	434.0 (305.7-606.7)	Falls	538.3 (380.5-741.6)	Other MSK	428.5 (291.1-600.7)	Neck pain	398.4 (275.3-559.6)	Diabetes mellitus	533.6 (354.5-742.3)	Depressive disorders	479.6 (337.7-653.1)
**6**	Neck pain	420.2 (290.2-589.8)	Depressive disorders	355.6 (251.0-483.7)	Depressive disorders	533.1 (376.3-725.1)	COPD	413.5 (333.9-491.5)	Depressive disorders	344.0 (243.8-471.7)	Anxiety disorders	506.4 (354.1-682.7)	Anxiety disorders	333.8 (233.9-451.2)
**7**	Neonatal disorders	362.1 (264.6-475)	Anxiety disorders	330.5 (234.3-445.3)	Neck pain	433.5 (299.7-608.9)	Falls	396.2 (280.8-543.3)	Anxiety disorders	331.7 (230.4-447.9)	Depressive disorders	442.8 (312.2-613.4)	Neck pain	332.3 (224.8-477.0)
**8**	Anxiety disorders	352.8 (250.2-469.3)	Hearing loss	277.1 (180.0-412.8)	Alcohol use disorders	355.2 (242.0-495.6)	Hearing loss	392.3 (269.6-552.3)	Hearing loss	312.3 (205.0-451.2)	Neonatal disorders	396.9 (285.2-530.2)	Dermatitis	310.3 (173.0-505.4)
**9**	Hearing loss	284.8 (187.6-417.1)	Oral disorders	267.1 (147.3-452.3)	Hearing loss	290.0 (195.7-416.2)	Diabetes mellitus	368.7 (247.8-513.4)	Neonatal disorders	296.1 (204.2-413.8)	Hearing loss	305.1 (200.5-444.8)	Asthma	303.8 (195.8-435.5)
10	Dermatitis	275.5 (153.8-448.2)	Dermatitis	247.8 (138.0-401.7)	Drug use disorders	275.5 (198.4-356.7)	Anxiety disorders	364.8 (256.5-485.4)	Asthma	294.6 (193.0-424.0)	Asthma	277.4 (179.9-400.3)	Hearing loss	275.1 (180.5-405.9)
**Total**	**All causes**	**10305.8 (7752.5-13361.3)**	**All causes**	**10194.9 (7618.5-13308.9)**	**All causes**	**10678.7 (8008.2-13757.8)**	**All causes**	**10458.5 (7891.6-13441)**	**All causes**	**9925.5 (7452.9-12872.8)**	**All causes**	**10763.9 (8094.8-13837.6)**	**All causes**	**9940.3 (7456.9-12950.4)**

Abbreviations: ASR=age-standardised rates. COPD=chronic obstructive pulmonary disease. Other MSK= other musculoskeletal disorders. UI=uncertainty intervals

Appendix table 5b. Age-standardised Years Lived with Disability (YLD) rates per 100,000 population of top-ten causes (Level 3) in the Nordic countries in 2017, females.

	**Females**													
	**The Nordic region**		**Denmark**		**Finland**		**Greenland**		**Iceland**		**Norway**		**Sweden**	
		**ASR (95% UI)**		**ASR (95% UI)**		**ASR (95% UI)**		**ASR (95% UI)**		**ASR (95% UI)**		**ASR (95% UI)**		**ASR (95% UI)**
**1**	Low back pain	1461.7 (1037.1-1990.9)	Low back pain	1714.1 (1209.9-2329.8)	Low back pain	1277.4 (905.7-1746.8)	Depressive disorders	1327.2 (929.5-1823.5)	Low back pain	1464.9 (1041.1-1934.2)	Low back pain	1420.1 (1008.3-1951.4)	Low back pain	1438.0 (1022.0-1961.0)
**2**	Headache disorders	1196.8 (788.7-1700.9)	Headache disorders	987.5 (659.9-1387.8)	Headache disorders	1178.8 (774.7-1685)	Low back pain	1207.4 (871.5-1625.2)	Headache disorders	1184.1 (783.0-1705.3)	Headache disorders	1282.1 (842.4-1831.6)	Headache disorders	1283.3 (852.8-1805.7)
**3**	Depressive disorders	812.0 (575.5-1104.1)	Anxiety disorders	610.7 (433.8-822.3)	Depressive disorders	933.7 (665.4-1264.9)	Headache disorders	1132.7 (743.3-1606.8)	Depressive disorders	633.7 (449.9-850.7)	Anxiety disorders	831.8 (581.9-1120.3)	Depressive disorders	942.1 (658.4-1278.7)
**4**	Anxiety disorders	611.0 (427.0-811.9)	Neck pain	598.8 (415.2-847.3)	Neck pain	704.4 (491.3-985.2)	Anxiety disorders	651.8 (468.3-866.2)	Anxiety disorders	611.2 (432.9-826.7)	Depressive disorders	701.0 (497.7-964.4)	Anxiety disorders	606.0 (419.7-813.5)
**5**	Neck pain	586.1 (407.6-826.7)	Depressive disorders	585.3 (418.8-791.6)	Neonatal disorders	568.1 (395.4-761.3)	Other MSK	548.0 (369.6-760.3)	Neck pain	558.2 (389.3-774.9)	Neck pain	682.0 (471.3-955.6)	Neck pain	463.1 (312.8-662.5)
**6**	Falls	431.0 (307.9-586.6)	Diabetes mellitus	513.8 (340.6-724.6)	Diabetes mellitus	468.2 (313.1-662.4)	COPD	504.7 (416.9-594.4)	Diabetes mellitus	412.4 (275.9-580.5)	Falls	462.8 (331.8-624.1)	Falls	447.8 (319.4-605.8)
**7**	Diabetes mellitus	426.2 (282.3-592.7)	Falls	365.0 (259.4-503.1)	Falls	442.3 (314.2-610.4)	Drug use disorders	395.3 (274.2-545.6)	Falls	357.5 (253.0-499.9)	Diabetes mellitus	382.1 (251.4-531.9)	Dermatitis	422.3 (238.2-685.5)
**8**	Dermatitis	361.5 (202.4-587.5)	Dermatitis	325.8 (182.0-530.1)	Anxiety disorders	402.2 (285.3-541.6)	Falls	391.2 (278.1-533.9)	Dermatitis	350.2 (195.4-574.1)	Oral disorders	333.6 (203.0-509.3)	Diabetes mellitus	376.2 (248.5-523.9)
**9**	Neonatal disorders	319.2 (234.4-419.4)	Oral disorders	318.6 (179.1-531.0)	Gyne-cological diseases	356.6 (253.0-486.3)	Neck pain	350.9 (243.9-488.1)	Asthma	346.1 (223.0-492.4)	Dermatitis	327.5 (184.4-529.0)	Asthma	315.2 (201.5-446.9)
**10**	Gyne-cological diseases	296.2 (208.4-405.2)	Other MSK	307.3 (207.1-434.0)	Dermatitis	320.7 (177.9-527.6)	Diabetes mellitus	333.0 (223.0-465.1)	COPD	337.2 (274.7-398.6)	Asthma	310.4 (200.7-443.0)	Gyne-cological diseases	310.5 (215.2-426.1)
**Total**	**All causes**	**11729.8 (8811.1-15194.9)**	**All causes**	**11322.1 (8511.4-14709.4)**	**All causes**	**11743.6 (8856.1-15139.2)**	**All causes**	**12516.8 (9448.2-15915.3)**	**All causes**	**11286.3 (8443-14524.8)**	**All causes**	**12017.5 (9032.7-15520.2)**	**All causes**	**11817.2 (8842.8-15266.8)**

Abbreviations: ASR=age-standardised rates. COPD=chronic obstructive pulmonary disease. Other MSK= other musculoskeletal disorders. UI=uncertainty intervals

Appendix table 6a. Age-standardised Disability-Adjusted Life-Years (DALY) rates per 100,000 population of top-ten causes (Level 3) in the Nordic countries in 2017, males.

	**Males**													
	**The Nordic region**		**Denmark**		**Finland**		**Greenland**		**Iceland**		**Norway**		**Sweden**	
		**ASR (95% UI)**		**ASR (95% UI)**		**ASR (95% UI)**		**ASR (95% UI)**		**ASR (95% UI)**		**ASR (95% UI)**		**ASR (95% UI)**
**1**	Ischaemic heart disease	1467.6 (1395.9-1575.7)	Low back pain	1448.0 (1021.6-1974.9)	Ischaemic heart disease	1989.5 (1829.5-2193.1)	Self-harm	3410.1 (3048.0-3804.2)	Ischaemic heart disease	1679.8 (1592.7-1780.5)	Ischaemic heart disease	1202.4 (1144.6-1276.5)	Ischaemic heart disease	1460.3 (1355.5-1590.1)
**2**	Low back pain	1185.8 (843.5-1607.8)	Ischaemic heart disease	1144.6 (1059.0-1246.2)	Low back pain	1112.0 (790.0-1522.8)	Ischaemic heart disease	2298.7 (2114.9-2465.4)	Low back pain	1208.0 (870.7-1607.8)	Low back pain	1176.5 (846.5-1608.9)	Low back pain	1078.9 (769.3-1456.9)
**3**	Diabetes	743.8 (547.3-978.1)	Diabetes	953.0 (713.9-1234.3)	Self-harm	912.0 (831.8-1006)	Lung cancer	1892.3 (1703.9-2064.5)	Self-harm	695.4 (639.3-754.0)	Falls	721.3 (560.5-927.7)	Self-harm	685.9 (634.3-745.9)
**4**	Self-harm	685.7 (653.1-721.4)	Lung cancer	783.1 (718.3-853.3)	Falls	809.4 (646.8-1007.1)	Stroke	1700.2 (1556.8-1849)	Diabetes	654.2 (471.1-882.3)	Diabetes mellitus	665.8 (485.4-872.2)	Diabetes mellitus	676.4 (505.4-886.4)
**5**	Falls	684.2 (537.1-877.1)	COPD	703.2 (642.2-760.9)	Neonatal disorders	788.9 (601.8-1008.8)	Neonatal disorders	1482.7 (1187.4-1823.3)	Lung cancer	630.6 (584.5-679.8)	Stroke	610.2 (549.0-670.6)	Falls	675.6 (517.7-874.7)
**6**	Stroke	626.6 (572.0-680.4)	Stroke	642.2 (579.8-704.4)	Alcohol use disorders	738.7 (616.2-893.7)	COPD	1048.3 (928.9-1169.0)	Headache disorders	627.9 (414.2-887.3)	Neonatal disorders	596.2 (477.7-732.0)	Headache disorders	665.4 (442.7-939.5)
**7**	Headache disorders	614.7 (408.9-861.6)	Alcohol use disorders	634.4 (551.4-735.6)	Diabetes	731.1 (522.5-983.0)	Low back pain	977.2 (695.5-1338.5)	Falls	621.9 (485.9-797.3)	Self-harm	596.0 (562.0-648.8)	Stroke	583.3 (523.0-641.1)
**8**	Neonatal disorders	604.2 (494.6-733.7)	Neonatal disorders	599.8 (474.9-747.4)	Stroke	700.4 (635.2-772.1)	Alcohol use disorders	891.1 (737.5-1102.4)	Stroke	518.1 (470.9-566.5)	Headache disorders	589.7 (393.2-830.6)	Neonatal disorders	512.9 (412.5-625.9)
**9**	Drug use disorders	540.0 (469.0-609.4)	Falls	543.2 (416.3-710.6)	Drug use disorders	636.9 (540.4-728.5)	Falls	804.9 (678.1-954.0)	COPD	513.8 (452.5-571.5)	Drug use disorders	564.2 (491.8-637.8)	Drug use disorders	503.3 (435.5-574.6)
**10**	Lung cancer	538.9 (515.2-567.1)	Headache disorders	540.5 (368.1-744.6)	Headache disorders	625.3 (414.1-883.1)	Depressive disorders	752.5 (520.3-1025.8)	Drug use disorders	429.3 (359.5-504.8)	COPD	546.5 (502.6-592.3)	Depressive disorders	479.6 (337.7-653.1)
**Total**	**All causes**	**19932.7 (17281.4-22954.3)**	**All causes**	**20696 (23821.3-17964.2)**	**All causes**	**21776.2 (19010.9-24970.2)**	**All causes**	**33101.4 (30182.3-36218.6)**	**All causes**	**19417.3 (16828.9-22390.5)**	**All causes**	**19646.9 (16916.5-22733)**	**All causes**	**18555.7 (15968.6-21426.8)**

Abbreviations: ASR=age-standardised rates. COPD=chronic obstructive pulmonary disease. UI=uncertainty intervals

Appendix table 6b. Age-standardised Disability-Adjusted Life-Years (DALY) rates per 100,000 population of top-ten causes (Level 3) in the Nordic countries in 2017, females.

	**Females**													
	**The Nordic region**		**Denmark**		**Finland**		**Greenland**		**Iceland**		**Norway**		**Sweden**	
		**ASR (95% UI)**		**ASR (95% UI)**		**ASR (95% UI)**		**ASR (95% UI)**		**ASR (95% UI)**		**ASR (95% UI)**		**ASR (95% UI)**
**1**	Low back pain	1461.7 (1037.1-1990.9)	Low back pain	1714.1 (1209.9-2329.8)	Low back pain	1277.4 (905.7-1746.8)	Self-harm	1359.8 (1229.9-1503.7)	Low back pain	1464.9 (1041.1-1934.2)	Low back pain	1420.1 (1008.3-1951.4)	Low back pain	1438.0 (1022.0-1961)
**2**	Headache disorders	1196.8 (788.7-1700.9)	Headache disorders	987.5 (659.9-1387.8)	Headache disorders	1178.8 (774.7-1685)	Depressive disorders	1327.2 (929.5-1823.5)	Headache disorders	1184.1 (783.0-1705.3)	Headache disorders	1282.1 (842.4-1831.6)	Headache disorders	1283.3 (852.8-1805.7)
**3**	Depressive disorders	812.0 (575.5-1104.1)	COPD	678.0 (614.1-745.1)	Depressive disorders	933.7 (665.4-1264.9)	COPD	1262.8 (1093.9-1428.5)	Depressive disorders	633.7 (449.9-850.7)	Anxiety disorders	831.8 (581.9-1120.3)	Depressive disorders	942.1 (658.4-1278.7)
**4**	Anxiety disorders	611.0 (427.0-811.9)	Lung cancer	665.6 (595.1-742.2)	Neonatal disorders	715.8 (541.2-910.9)	Lung cancer	1244 (1080.1-1421.5)	Anxiety disorders	611.2 (432.9-826.7)	Depressive disorders	701.0 (497.7-964.4)	Ischaemic heart disease	642.6 (587.7-706.1)
**5**	Ischaemic heart disease	588.3 (553.9-634.3)	Diabetes mellitus	637.3 (465.5-845.4)	Neck pain	704.4 (491.3-985.2)	Low back pain	1207.4 (871.5-1625.2)	Neck pain	558.2 (389.3-774.9)	Neck pain	682.0 (471.3-955.6)	Anxiety disorders	606.0 (419.7-813.5)
**6**	Neck pain	586.1 (407.6-826.7)	Anxiety disorders	610.7 (433.8-822.3)	Ischaemic heart disease	681.2 (622.2-751.1)	Headache disorders	1132.7 (743.3-1606.8)	COPD	530.4 (463.8-595.2)	Falls	534.8 (403.2-696.0)	COPD	509.2 (452.2-568.0)
**7**	Neonatal disorders	508.7 (411.6-614.2)	Neck pain	598.8 (415.2-847.3)	Alzheimer's disease	538.4 (497.4-582.2)	Neonatal disorders	1091.6 (869.2-1362.6)	Lung cancer	501.4 (457.2-549.2)	Ischaemic heart disease	495.4 (470.7-525.8)	Falls	500.9 (374.0-661.4)
**8**	Diabetes mellitus	504.5 (360.0-670.0)	Depressive disorders	585.3 (418.8-791.6)	Falls	527.1 (397.2-692.7)	Stroke	1029 (909.5-1162.7)	Diabetes mellitus	444.0 (307.4-611.4)	COPD	465.1 (429.3-502.0)	Breast cancer	467.5 (426.7-513.1)
**9**	Falls	494.9 (370.8-649.8)	Breast cancer	547.8 (492.2-610.0)	Stroke	524.4 (466.1-583.1)	Ischaemic heart disease	802.8 (704.9-931.8)	Ischaemic heart disease	442.6 (409.2-476.9)	Stroke	461.1 (415.3-509.1)	Neck pain	463.1 (312.8-662.5)
**10**	Stroke	480.1 (433.9-525.1)	Neonatal disorders	501.2 (407.2-618.3)	Diabetes mellitus	513.4 (358.2-706.9)	Anxiety disorders	651.8 (468.3-866.2)	Neonatal disorders	419.0 (319.3-542.9)	Diabetes mellitus	447.8 (315.9-598.8)	Stroke	458.3 (409.9-509.7)
**Total**	**All causes**	**17762.2 (14873.2-21228.4)**	**All causes**	**18330.9 (15503.9-21650.3)**	**All causes**	**17524.3 (14522.1-20886.8)**	**All causes**	**26666.6 (23478.4-30218.8)**	**All causes**	**16074.1 (13216.4-19240.8)**	**All causes**	**17794.2 (14811.1-21288.1)**	**All causes**	**17563.1 (14584.4-20977.3)**

Abbreviations: ASR=age-standardised rates. COPD=chronic obstructive pulmonary disease. UI=uncertainty intervals.

Appendix table 7a. Number of disability-adjusted life-years (DALY) and percent of total DALYs attributed to top-ten risk factors1 in the Nordic countries, males.

Males

The Nordic Region	DALYs	%[2]	Denmark	DALYs	%[2]	Finland	DALYs	%[2]
Smoking	465374	12.5	Smoking	136707	16.4	Alcohol use	93088	11.0
High glucose	413185	11.1	High glucose	101682	12.2	High BP	107867	12.7
High BP	390873	10.5	Alcohol use	82050	9.9	High glucose	94118	11.1
Alcohol use	288341	7.7	High BP	74728	9.0	Smoking	93797	11.0
High BMI	313619	8.4	High BMI	66755	8.0	High BMI	78410	9.2
High cholest	185707	5.0	High cholest	31225	3.8	High cholest	52214	6.1
Drug use	94728	2.5	Drug use	19219	2.3	Drug use	24558	2.9
Low whole grains	136678	3.7	Low whole grains	28481	3.4	Low whole grains	34482	4.1
Low nuts+seeds	89515	2.4	Part Matt Poll	24845	3.0	Low nuts+seeds	27059	3.2
Low fruits	72658	1.9	Birth weight+gest	7242	0.9	Low fruits	23077	2.7
**All risk factors**	**1697476**	**45.5**	**All risk factors**	**407785**	**49.0**	**All risk factors**	**410104**	**48.2**

Greenland	DALYs	%[2]	Iceland	DALYs	%[2]
Smoking	2002	18.3	Smoking	4818	11.8
Alcohol use	887	8.1	High glucose	4143	10.1
High BP	851	7.8	High BMI	3909	9.6
High glucose	721	6.6	High BP	3713	9.1
High BMI	770	7.0	Alcohol use	2005	4.9
Birth weight+gest	279	2.5	High cholest	2302	5.6
High cholest	476	4.4	Low whole grains	1466	3.6
Drug use	332	3.0	Drug use	931	2.3
Low whole grains	368	3.4	Low nuts+seeds	1125	2.8
Part Matt Poll	312	2.9	Occup Ergon	639	1.6
**All risk factors**	**5341**	**48.9**	**All risk factors**	**17457**	**42.7**

Norway	DALYs	%[2]	Sweden	DALYs	%[2]
Smoking	83712	12.3	High glucose	148086	11.3
High glucose	64436	9.5	Smoking	144338	11.0
High BP	59854	8.8	High BP	143860	10.9
High BMI	51456	7.6	High BMI	112319	8.5
Alcohol use	32538	4.8	Alcohol use	77771	5.9
High cholest	30057	4.4	High cholest	69434	5.3
Drug use	18399	2.7	Drug use	31289	2.4
Low whole grains	21057	3.1	Low whole grains	50825	3.9
High proc meat	13944	2.1	Low nuts+seeds	32398	2.5
Low nuts+seeds	13141	1.9	Diet high in sodium	29108	2.2
**All risk factors**	**280884**	**41.4**	**All risk factors**	**575905**	**43.8**

[1]Top ten risk factors ranked according to ranking for age-standardised rates, see Figure 3.
[2]Percent of total number of all cause DALYs attributed to all included risk factors in GBD.

Abbreviations: ASR=age-standardised rates. DALYs=disability-adjusted life-years. Birth weight+gest=low birth weight and short gestation. High BMI=high body-mass index. High BP=high systolic blood pressure. High cholest=high low-density lipoprotein cholesterol. High glucose=high fasting plasma glucose. High Proc Meat=diet high in processed meat. Low fruits=diet low in fruits. Low nuts+seeds=diet low in nuts and seeds. Low whole grains=diet low in whole grains. Occup Ergon=occupational ergonomic factors. Part Matt Poll=particulate matter pollution. UI=uncertainty intervals.

Appendix table 7b Number of disability-adjusted life-years (DALY) and percent of total DALYs attributed to top-ten risk factors1 in the Nordic countries, females.

**Females**																				
**The Nordic Region**			**Denmark**			**Finland**			**Greenland**			**Iceland**			**Norway**			**Sweden**		
	**DALYs**	**%[2]**		**DALYs**	**%[2]**		**DALYs**	**%[2]**		**DALYs**	**%[2]**		**DALYs**	**%[2]**		**DALYs**	**%[2]**		**DALYs**	**%[2]**
Smoking	349639	9.9	Smoking	108166	13.9	High BMI	63851	8.5	Smoking	1183	15.5	Smoking	2945	8.5	Smoking	55241	8.6	Smoking	137680	10.6
High glucose	313350	8.9	High glucose	76643	9.8	High glucose	70967	9.4	High BMI	483	6.3	High BMI	2531	7.3	High glucose	48735	7.6	High glucose	114221	8.8
High BMI	253715	7.2	High BMI	53389	6.8	High BP	73383	9.7	High BP	367	4.8	High glucose	2430	7.0	High BMI	40097	6.2	High BMI	93364	7.2
High BP	278228	7.9	High BP	52211	6.7	Smoking	44423	5.9	High glucose	353	4.6	High BP	1865	5.4	High BP	43004	6.7	High BP	107399	8.3
Alcohol use	80362	2.3	Alcohol use	26204	3.4	Alcohol use	14225	1.9	Alcohol use	303	4.0	Occup Ergon	554	1.6	High cholest	18389	2.9	Alcohol use	32832	2.5
High cholest	113904	3.2	Birth weight+gest	6373	0.8	High cholest	29941	4.0	Birth weight+gest	193	2.5	High cholest	892	2.6	Low whole grains	13876	**2.2**	High cholest	45682	3.5
Low whole grains	88172	2.5	Low whole grains	18588	2.4	Low whole grains	21053	2.8	Drug use	196	2.6	Low whole grains	689	2.0	Occup Ergon	8151	**1.3**	Low whole grains	33818	2.6
Occup Ergon	40166	1.1	Part Matt Poll	19607	2.5	Drug use	7773	1.0	Part Matt Poll	173	2.3	Birth weight+gest	307	0.9	Alcohol use	6512	1.0	Occup ergon	15045	1.2
Birth weight+gest	24171	0.7	High cholest	18853	2.4	Occup Ergon	7412	1.0	High cholest	147	1.9	Drug use	352	1.0	Birth weight+gest	4451	0.7	Birth weight+gest	8388	0.6
Imp kid	64768	1.8	Occup ergon	8949	1.1	Birth weight+gest	4459	0.6	Low whole grains	149	1.9	Alcohol use	287	0.8	Drug use	6257	1.0	Imp kid	25110	1.9
**All risk factors**	**1280968**	**36.4**	**All risk factors**	**309094**	**39.6**	**All risk factors**	**269403**	**35.7**	**All risk factors**	**3134**	**40.9**	**All risk factors**	**10925**	**31.6**	**All risk factors**	**209741**	**32.6**	**All risk factors**	**478671**	**36.8**

[1]Top ten risk factors ranked according to ranking for age-standardised rates, see Figure 3.

[2]Percent of total number of all cause DALYs attributed to all included risk factors in GBD.

Abbreviations: ASR=age-standardised rates. DALYs=disability-adjusted life-years. Birth weight+gest=low birth weight and short gestation. High BMI=high body-mass index. High BP=high systolic blood pressure. High cholest=high low-density lipoprotein cholesterol. High glucose=high fasting plasma glucose. Imp Kidney=impaired kidney function. Low whole grains=diet low in whole grains. Occup Ergon=occupational ergonomic factors. Part Matt Poll=particulate matter pollution. UI=uncertainty intervals.

Appendix table 8a. Age-standardised disability-adjusted life-years (DALY) rates per 100,000 population of top-ten risk factors (Level 3) in the Nordic countries in 2017, males.

**Males**														
	**The Nordic region**		**Denmark**		**Finland**		**Greenland**		**Iceland**		**Norway**		**Sweden**	
		**ASR (95% UI)**		**ASR (95% UI)**		**ASR (95% UI)**		**ASR (95% UI)**		**ASR (95% UI)**		**ASR (95% UI)**		**ASR (95% UI)**
**1**	Smoking	1966.7 (1796.2-2136.4)	Smoking	2717.3 (2459.8-2973.2)	Alcohol use	2531.1 (1982.9-3192.4)	Smoking	5216.2 (4621.1-5771.4)	Smoking	1967.5 (1768.1-2161.9)	Smoking	1990.0 (1813.2-2193.9)	High glucose	1613.5 (1240.0-2013.4)
**2**	High glucose	1770.5 (1387.5-2206)	High glucose	2088.6 (1652.2-2595.5)	High BP	2116.5 (1883.3-2350)	Alcohol use	2482.7 (1579.2-3410)	High glucose	1701.5 (1290.1-2164.2)	High glucose	1557.0 (1212.3-1930.1)	Smoking	1565.0 (1391-1735.9)
**3**	High BP	1631.5 (1455.5-1799.7)	Alcohol use	1955.9 (1456-2505.8)	High glucose	1909.1 (1450.5-2460.4)	High BP	2340.3 (2024.6-2659.4)	High BMI	1674.6 (1075.1-2337.6)	High BP	1412.9 (1250.4-1557.8)	High BP	1540.7 (1346.3-1732.6)
**4**	Alcohol use	1579.7 (1277.8-1926.3)	High BP	1482.3 (1304.1-1655.5)	Smoking	1878.1 (1678.8-2078.9)	High glucose	2087.1 (1569.7-2711.9)	High BP	1504.2 (1318.0-1694.3)	High BMI	1301.3 (814.6-1860.5)	High BMI	1350.0 (811.2-1943)
**5**	High BMI	1443.5 (893.1-2053.7)	High BMI	1454.9 (875.3-2104.3)	High BMI	1702.3 (1061.8-2412.6)	High BMI	1952.2 (1109.9-2883.7)	Alcohol use	991.9 (619.9-1427.0)	Alcohol use	976.4 (686.2-1301.4)	Alcohol use	1160.9 (807.7-1587.3)
**6**	High cholest	812.3 (659.8-971.0)	High cholest	650.6 (524.3-791.7)	High cholest	1068.8 (852.6-1295.1)	Birth Weight+Gest	1348.6 (1073.3-1666.4)	High cholest	952.8 (781.5-1133.7)	High cholest	732.5 (599.7-879.0)	High cholest	787.0 (627.8-958.2)
**7**	Drug use	665.8 (587.9-745.9)	Drug use	618.8 (538.3-707.3)	Drug use	849.3 (739.5-965.3)	High cholest	1216.7 (990.7-1472.7)	Low whole grains	618.6 (430.4-849.7)	Drug use	649.7 (573.5-729.2)	Drug use	601.4 (525.6-680.2)
**8**	Low whole grains	609.7 (429.5-818.8)	Low whole grains	605.6 (431.4-815.5)	Low whole grains	726.0 (500.4-979.0)	Drug use	1038.3 (823.4-1303.7)	Drug use	517.9 (442.9-598.5)	Low whole grains	518.9 (364.7-703.3)	Low whole grains	587.3 (412.7-792.1)
**9**	Low nuts+ seeds	393.6 (259.8-543.7)	Pollution	507.0 (346.7-664.1)	Low nuts+ seeds	556.4 (366.9-757.6)	Low whole grains	948.6 (658.1-1266.0)	Low nuts+ seeds	470.7 (313.9-647.8)	High Proc Meat	352.7 (166.1-515.3)	Low nuts+ seeds	367.8 (238.1-512.0)
**10**	Low fruits	324.5 (173.8-509.6)	Birth Weight+Gest	424.5 (330.4-537.4)	Low fruits	482.5 (276.4-729.5)	Pollution	843.4 (303.0-1299.1)	Occup Ergon	325.7 (234.1-431.4)	Low nuts+seeds	322.8 (212.5-445.7)	Diet high in sodium	316.7 (37.2-664.3)
**Total**	**All risk factors**	**8272.6 (7459.2-9144.8)**	**All risk factors**	**9341.9 (8394.3-10411.1)**	**All risk factors**	**9592.4 (8618.4-10706.9)**	**All risk factors**	**15526.3 (14226.4-16887.2)**	**All risk factors**	**7747.8 (6954.0-8665.6)**	**All risk factors**	**7511.1 (6719.8-8373.7)**	**All risk factors**	**7286.1 (6494.1-8174.9)**

Abbreviations: ASR=age-standardised rates. DALYs=disability-adjusted life-years. Birth weight+gest=low birth weight and short gestation. High BMI=high body-mass index. High BP=high systolic blood pressure. High cholest=high low-density lipoprotein cholesterol. High glucose=high fasting plasma glucose. Low fruits=diet low in fruit. Low nuts+seeds=diet low in nuts and seeds. Low whole grains=diet low in whole grains. Occup Ergon=occupational ergonomic factors. Part Matt Poll=particulate matter pollution. UI=uncertainty intervals.

Appendix table 8b. Age-standardised disability-adjusted life-years (DALY) rates per 100,000 population of top-ten risk factors (Level 3) in the Nordic countries in 2017, females.

**Females**														
	**The Nordic region**		**Denmark**		**Finland**		**Greenland**		**Iceland**		**Norway**		**Sweden**	
		**ASR (95% UI)**		**ASR (95% UI)**		**ASR (95% UI)**		**ASR (95% UI)**		**ASR (95% UI)**		**ASR (95% UI)**		**ASR (95% UI)**
**1**	Smoking	1355.2 (1207.9-1510.6)	Smoking	1944.5 (1731.4-2174.6)	High BMI	1133.1 (755.5-1587.9)	Smoking	3685.8 (3192.4-4203.4)	Smoking	1156.6 (996.4-1324.7)	Smoking	1238.2 (1079.7-1405.7)	Smoking	1398.4 (1224.9-1575.4)
**2**	High glucose	1086.2 (815.1-1398.9)	High glucose	1327.1 (982.2-1730.4)	High glucose	1090.9 (805.5-1424.3)	High BMI	1505.5 (1003.6-2040.3)	High BMI	1019.5 (687.1-1413.5)	High glucose	957.9 (716.2-1245.7)	High glucose	1013.9 (756.1-1317.7)
**3**	High BMI	1024.3 (683.4-1433.9)	High BMI	1062.2 (705.5-1485.0)	High BP	997.9 (865.5-1120.7)	High BP	1200.6 (1026.5-1404.3)	High glucose	870.8 (631.5-1144.6)	High BMI	910.2 (595.1-1287.6)	High BMI	989.8 (645.2-1398.6)
**4**	High BP	873.6 (761.5-973.5)	High BP	821.5 (716.2-924.7)	Smoking	819.6 (700.4-951.7)	High glucose	1159.8 (831.8-1536.2)	High BP	605.5 (518.2-689.2)	High BP	779.2 (685-873.4)	High BP	874.2 (754.2-996.2)
**5**	Alcohol use	473.2 (319.3-644.0)	Alcohol use	619.8 (358.2-946.2)	Alcohol use	497.0 (309.2-715.6)	Alcohol use	998.2 (671.8-1372.8)	Occup Ergon	293.7 (207.9-392.9)	High cholest	327.2 (251.9-414.5)	Alcohol use	487.0 (275.2-753.9)
**6**	High cholest	356.2 (271.9-454.7)	Birth Weight+Gest	371.4 (300.3-459.9)	High cholest	397 (295.5-510.2)	Birth Weight+Gest	985.9 (777.7-1240.6)	High cholest	283.5 (219.5-354.3)	Low whole grains	288.9 (206.7-388.3)	High cholest	378.8 (286.8-483.3)
**7**	Low whole grains	329.9 (236.0-444.5)	Low whole grains	347.8 (247.9-472.3)	Low whole grains	350.9 (248-476.8)	Drug use	700.2 (540.2-886.6)	Low whole grains	260.5 (183.3-354.1)	Occup Ergon	277.6 (198.0-391.5)	Low whole grains	328.6 (237.2-443.2)
**8**	Occup Ergon	271.7 (192.4-373.3)	Part Matt Poll	345.2 (227.3-458.3)	Drug use	247.4 (211.4-289.5)	Part Matt Poll	552.7 (173.1-872.1)	Birth Weight+Gest	234.7 (180.7-310.4)	Alcohol use	270.7 (130.3-431.2)	Occup Ergon	274.6 (191.1-385.5)
**9**	Birth Weight+Gest	271.7 (227.0-318.3)	High cholest	295.1 (225.6-377.0)	Occup Ergon	247.1 (171.7-337.1)	High cholest	467.3 (354.2-602.1)	Drug use	194.1 (163.1-227.4)	Birth Weight+Gest	240.1 (196.8-288.1)	Birth Weight+Gest	244.4 (195.0-292.3)
**10**	Imp Kidney	210.5 (182.5-239.8)	Occup Ergon	284.4 (200.9-392.5)	Birth Weight+Gest	246.5 (197.6-306.7)	Low whole grains	467.3 (335.7-616.2)	Alcohol use	182.7 (62.0-323.3)	Drug use	215.2 (189.0-243.7)	Imp Kidney	211.2 (180.5-242.8)
**Total**	**All risk factors**	**5381.5 (4740.6-6136.3)**	**All risk factors**	**6244 (5511.9-7068.4)**	**All risk factors**	**5117.7 (4405.9-5925.1)**	**All risk factors**	**10598 (9512.4-11761.2)**	**All risk factors**	**4488.7 (3859-5222.5)**	**All risk factors**	**4943.1 (4312.5-5647)**	**All risk factors**	**5265.6 (4587.8-6045.5)**

Abbreviations: ASR=age-standardised rates. DALYs=disability-adjusted life-years. Birth weight+gest=low birth weight and short gestation. High BMI=high body-mass index. High BP=high systolic blood pressure. High cholest=high low-density lipoprotein cholesterol. High glucose=high fasting plasma glucose. Imp Kidney=impaired kidney function. Low whole grains=diet low in whole grains. Occup Ergon=occupational ergonomic factors. Part Matt Poll=particulate matter pollution. UI=uncertainty intervals.
